# The Effect of Vaccination Status on Total Lymphocyte Count in Horses Affected by Equine Herpes Virus-1 Myeloencephalopathy

**DOI:** 10.3390/ani15071019

**Published:** 2025-04-01

**Authors:** María de la Cuesta-Torrado, Valentina Vitale, Ana Velloso Alvarez, Patricia Neira-Egea, Clairianne Diss, Juan Cuervo-Arango

**Affiliations:** 1 Department of Animal Medicine and Surgery, Universidad Cardenal Herrera-CEU, CEU Universities, 46115 Alfara del Patriarca, Valencia, Spain; valentina.vitale@uchceu.es (V.V.); ana.vellosoalvarez@uchceu.es (A.V.A.); clairianne.diss@outlook.com (C.D.); juan.cuervo@uchceu.es (J.C.-A.); 2 Veterinary Teaching Hospital, Universidad Cardenal Herrera-CEU, CEU Universities, 46115 Alfara del Patriarca, Valencia, Spain; patricia.neiraegea@uchceu.es; 3 Clinique Equine de Provence, 13760 Saint-Cannat, France

**Keywords:** equine herpesvirus 1, myeloencephalopathy, severity, lymphocytes, vaccination

## Abstract

Equine herpesvirus 1-induced myeloencephalopathy has a significant impact on the equine industry. Nevertheless, the clinical variables that may affect the severity of the disease are still under investigation. The objective of this research is studying the relationship between the level of lymphopenia and vaccination status with the severity of the disease in horses at an event, considering whether they had been correctly vaccinated or not prior to exposure to EHV-1. Ten horses were admitted to a veterinary teaching hospital following an equine herpesvirus myeloencephalopathy outbreak during an international show jumping competition in Spain. Data were collected from passport vaccination records, daily analyses, and the clinical histories of the affected horses. Correctly vaccinated horses had a significantly longer hospitalization duration (6/10, 15.5 ± 1.2 days) compared to incorrectly vaccinated horses (4/10, 12.5 ± 1.2 days; *p* = 0.01). Lymphopenia (<1.6 × 10^3^ lymphocytes/µL) was the most common leukogram abnormality. Correctly vaccinated horses demonstrated a higher lymphocyte count compared to incorrectly vaccinated horses within 24 h of admission (*p* < 0.01). This difference remained significant from days 1 to 4 and on day 6 post-admission (*p* =0.03). This study found that lymphopenia is a common leukogram alteration in equine herpesvirus 1-infected horses, and horses correctly vaccinated prior to an equine herpesvirus myeloencephalopathy outbreak tend to have a longer hospitalization time. Correctly vaccinated horses exhibited higher lymphocyte counts during the first 24 h and throughout hospitalization compared to incorrectly vaccinated horses. The immune system could play a relevant role in influencing the severity of equine herpesvirus myeloencephalopathy outbreaks, highlighting the need for further studies in this area.

## Simple Summary

Equine herpesvirus 1-induced myeloencephalopathy significantly affects the equine industry, yet factors influencing disease severity remain under investigation. The objective of this research is studying the relationship between lymphopenia and vaccination status in horses exposed to EHV-1 at an international show jumping competition in Spain. Ten horses admitted to a veterinary teaching hospital during an outbreak were analyzed using vaccination records, clinical histories, and daily blood tests. Correctly vaccinated horses had longer hospitalization than incorrectly vaccinated ones. Lymphopenia was the most frequent leukogram abnormality. Correctly vaccinated horses had higher lymphocyte counts within 24 h of admission, with this difference remaining significant from days 1 to 4 and on day 6. Findings suggest that correctly vaccinated horses experience prolonged hospitalization but maintain higher lymphocyte levels, indicating a potential immune response effect. Lymphopenia is a common alteration in EHV-1-infected horses, reinforcing the need for further research on the immune system’s role in disease severity.

## 1. Introduction

Equine herpesvirus type 1 (EHV-1) represents one of the most important and ubiquitous infectious diseases in the equine industry [1]. This viral pathogen can cause outbreaks of respiratory disease, abortion, or myeloencephalopathy (EHM), especially in the competition world, which can cause significant economic losses [2,3]. Following primary exposure, EHV-1 establishes a lifelong latent infection in the trigeminal ganglia or in peripheral blood mononuclear cells (PBMCs) [4]. Horses with latent EHV-1 infections are susceptible to reactivation and replication of the virus, particularly after exposure to stress factors [4]. Once the pathogen enters the host, a primary infection of the respiratory tract occurs, followed by a primary viremia and a possible second leucocyte-assisted viremia, which leads to dissemination of the virus to other organs, like the central nervous system (CNS) [5]. Once the pathogen reaches the CNS, the virus infects the endothelial cells of its arterioles and venules. It then leads to vasculitis, thrombosis, hypoxia, and ischemic damage causing neurological signs [6].

The risk factors described for the development and severity of EHM are highly diverse [3,7] including viral, environmental [8], and host factors [8,9,10,11]. Among them, the immunological status, particularly in relation to vaccination, has been proposed as a relevant factor [12]. Vaccination against EHV-1 has been the focus of extensive research, although its effectiveness and effects are still doubted [3]. Currently, there are two types of vaccines available commercially [13], a modified live vaccine (Rhinomune, Boehringer Ingelheim) and an inactivated (killed) vaccine from Zoetis with two options: EHV-1 (Pneumabort-K^®^+1b) and a combined vaccine for EHV-1 and 4 (Equip^®^). Both live and inactivated vaccines have been shown to significantly suppress EHV-1 disease and reduce nasal viral shedding, in a study conducted to evaluate the protective efficacy of commercially available vaccines. However, the duration of viremia was significantly reduced only in the group that received the inactivated vaccine [14]. It has been proved that vaccination reduces the incidence of abortion by EHV-1, but it minimally reduces the incidence of clinical signs associated with EHV-1 infection, and less is known regarding EHM [15,16]. Consequently, there is an important need for additional research to clarify the role of vaccination and immune response in EHM pathogenesis.

Research on recent EHM outbreaks has provided valuable information about factors that could affect the severity of the disease, such as the grade of ataxia on admission to the hospital or the development of systemic complications [17,18,19]. Lymphopenia has recently been described as the most frequent laboratory abnormality in horses with EHM [17], similar to what has been reported for other viral diseases such as equine coronavirus [20]. However, to date, extensive information on this variable and its relationship with the severity of EHM is not available. In the last few decades, the incidence of EHM outbreaks has increased worldwide [21,22,23]; in February 2023, the Valencian Community (Spain) experienced one outbreak of EHV-1 caused by the N virus strain (point mutation EHV-1 A2254) [24]. The study of horses affected by EHV-1 provided valuable data to expand the available knowledge on clinical variables influencing the severity of EHM.

Therefore, the objectives of this study were the following: (1) to assess the level of lymphopenia in horses affected by EHM and its relationship with the severity of the disease; (2) to study the relationship of the pre-outbreak vaccination status of affected horses with the severity of the disease; and (3) to evaluate the impact of vaccination status on total lymphocyte count. The hypothesis is that vaccination status, as well as lymphocyte count at disease onset, would influence the severity of EHM, demonstrating an interaction exists between these two factors.

## 2. Material and Methods

### 2.1. Inclusion Criteria

The experimental design of this research consisted of a retrospective study of clinical data from horses admitted to a veterinary teaching hospital (VTH), from one EHM outbreak occurring in the Valencian Community (Spain), in February 2023, during an international show jumping competition event, approved by the International Equestrian Federation (FEI). Horses with fever (rectal temperature ≥ 38.2 °C) during the sporting event, those that had ataxia grades ≥ 3/5 according to the modified scale of de Lahunta and Mayhew [25], showing altered mental status, and/or cranial nerve dysfunction were referred to the VTH. Additionally, horses with lower-grade ataxia (0–2) but with signs of colic were also included in the admission criteria.

### 2.2. Clinical Parameters on Admission and Hospitalization

During admission, an extended anamnesis was collected for each horse, thanks to the owners and official veterinarians of the competition. The information collected included breed, sex, age, vaccination status, clinical signs, and treatments received prior admission. During hospitalization, fever (rectal temperature ≥ 38.2 °C), ataxia, and central neurological signs were evaluated and registered daily to effectively assess the horses’ evolution. The therapeutic management of the referred horses is detailed in Supplementary Material S1. The decision to discharge the animals was based on the following criteria: no central neurological signs, improvement in the degree of ataxia by up to 2 points from the time of admission, and resolution of the complications developed during the disease. The variables used to assess the severity of the horses hospitalized were used in other similar studies: (1) days of hospitalization [26] (in case horses needed to stay for longer duration due to transportation arrangements, these days were not considered); and (2) complications associated with the disease [17].

### 2.3. Laboratory Parameters on Admission and Hospitalization

As the horses were admitted to the VTH, a blood sample was obtained from every horse to perform a complete blood analysis, which was performed using an automated ADVIA^®^ 2120i analyzer (ADVIA^®^ 2120i Siemens Healthcare Diagnostics Inc, Erlangen, Germany). Lymphocyte values were studied during the first 24 h of the horses’ admission and first week during the hospitalization time, reference values: 1600–5800 cells/µL [27,28].

Disease diagnosis was based on a positive EHV-1 PCR result from at least one of the nasopharyngeal swabs or blood samples in EDTA tubes taken during competition, or at the hospital. Samples were analyzed at two official national laboratories in Spain (National Reference Laboratory (Algete, Spain) and VISAVET Health Surveillance Centre (Madrid, Spain). All horses admitted with fever or neurological signs were considered suspected of EHM for treatment and isolation purposes until the next PCR was per-formed, as they originated from the outbreak location. Affected horses were considered positive only if they had a positive result on any of the tests, and all admitted horses were confirmed with at least one positive PCR result.

### 2.4. Vaccination Status of Referred Horses

Vaccination records for the year before the outbreak were obtained for all horses by checking the horses’ passport vaccination records. Appropriate vaccination status consisted of two initial doses of the EHV-1 vaccine at a 4-week interval, followed by at least one booster administration yearly. The horses that followed this vaccination schedule were included in the “correctly vaccinated group”. The passports of the horses that did not show this vaccination schedule were identified, and their owners were contacted by telephone and confirmed verbally the vaccination schedule recorded in the passport, as has been performed in previous studies [17,29]. These horses were included in the “incorrectly vaccinated group”.

### 2.5. Comorbidities Developed During Hospitalization

Any complication developed during hospitalization was registered. For data analyses, they were grouped into 3 categories: urinary problems (cystitis, urinary incontinence), clinical signs of vasculitis (limb edema, myocarditis, thrombus, petechiae), and other complications (colic, musculoskeletal, ophthalmological problems).

### 2.6. Statistical Analyses

Data collected from the horses included in the study (sex, age, breed, presence of neurological signs and other systemic signs, days of hospitalization, total lymphocyte count, and vaccination status) were evaluated using descriptive statistics. Continuous data were tested for normality using the Shapiro–Wilk test. The differences between sex and the development of vascular complications according to the correctly vaccinated or incorrectly vaccinated animal group were evaluated using Fisher’s exact test and with unpaired *t*-test for age distribution in both groups. The difference in the mean duration of hospitalization between correctly vaccinated and incorrectly vaccinated horses and between horses with or without lymphopenia 24 h after admission were tested by unpaired *t*-test.

The association between the percentage of horses with lymphopenia within 24 h after admission and the likelihood of developing complications was determined by Fisher’s exact test. A general linear model of variance with repeated measures to account for autocorrelation between individuals was performed to analyze the evolution of the lymphocyte count over time based on vaccination status. If a significant difference in group or group by day interaction was observed, an unpaired *t*-test was used to determine the difference in the lymphocyte count between correctly vaccinated and incorrectly vaccinated horses in each day. All the analyses were performed using a commercially available software (IBM SPSS Statistics software version 20) with a *p*-value for significance set at *p* < 0.05.

## 3. Results

### 3.1. Outbreak Details for Inclusion Criteria

Horses with EHM from the sporting event located in Oliva, Spain, which presented fever, ataxia, and/or colic signs, were admitted to the VTH. Out of 82 horses from the EHV-1 outbreak, 10 horses were affected by EHM and referred to the VTH. The morbidity was 12% (10/82).

### 3.2. Clinical Parameters During Admission and Hospitalization

All 10 horses referred to the VTH were Central Europeans breeds: Belgian (n = 1), Warmblood (n = 2), Holsteiner (n = 1), Koninklijk Warmbloed Paardenstamboek Neder-land (KWPN) (n = 1), Oldenburg (n = 1), and Selle Français (n = 4). Of the 10 horses referred, 6 were geldings and 4 were mares. The average age was 9.3 ± 2.0 years old (from 6 to 12). The horses’ country of origin was Belgium (n = 1), France (n = 4), United Kingdom (n = 1), Netherlands (n = 1), Norway (n = 1), and Ireland (n = 2). No horse received any medication prior to hospital admission.

The median ataxia grade upon admission was 3/5 (minimum 0, maximum of 4). Initially, only three horses arrived with fever (30% (3/10)), while the others developed fever in the following days. The fatality rate of the outbreak was 0% (0/10) since no horse was euthanized either during hospitalization or 14 months following hospital discharge. The mean hospitalization time was 14.1 ± 2.2 days.

### 3.3. Laboratory Parameters During Admission and Hospitalization

During the hospitalization period, all 10 horses tested positive for EHV-1 in at least one PCR test. Lymphopenia (<1600 cells/µL) was the most common abnormal finding on the leukogram within the first 24 h from admission in 7/10 cases (70%). The remaining horses had lymphocyte count values within the reference range. The mean count of lymphocytes was 1.32 ± 0.33 × 10^3^ cells/µL within the first 24 h from admission and 1.7 ± 0.5 cells 10^3^ cells/µL, at the time of discharge. The association between longer hospitalization times and lymphopenia did not show a significant difference (*p* > 0.05; unpaired *t*-test).

### 3.4. Vaccination Status of Admitted Horses

Six out of the ten hospitalized horses (60%) were considered correctly vaccinated. All these six horses had been vaccinated with an inactivated vaccine against EHV-1 and 4 (Equip^®^, Zoetis, Berlin, Germany). The four horses classified as incorrectly vaccinated had been vaccinated with the same vaccine, but without an adequate vaccination schedule. The age and female/gelding proportion was similar (*p* > 0.1; unpaired *t*-test and Fisher’s exact test, respectively) in the correctly vaccinated (10 ± 2 years and 50/50% mare/gelding) and incorrectly vaccinated horses (8.2 ± 1.5 and 25/75% mare/gelding).

### 3.5. Comorbidities During Hospitalization

Only two horses developed systemic signs of vasculitis (limb edema and thrombus in jugular vein), while no horse developed urinary or other types of relevant complications.

### 3.6. Impact of Vaccination on Clinical Parameters and Disease Severity

Three of the six correctly vaccinated horses (50%) presented fever at admission, while all incorrectly vaccinated horses (n = 4) were admitted with a normal rectal temperature. The median ataxia score (3) was similar (*p* > 0.1; Mann–Whitney non-parametric test) in correctly vaccinated and incorrectly vaccinated horses. Correctly vaccinated horses were more likely (*p* = 0.01; unpaired *t*-test) to have a longer hospitalization time (6/10, 15.5 ± 1.2 days) than incorrectly vaccinated horses (4/10, 12.5 ± 1.2 days; Table 1). Two correctly vaccinated horses (33.3%) developed systemic signs of vasculitis, while none of the incorrectly vaccinated horses showed any systemic complication (*p* > 0.1; Fisher’s exact test).

### 3.7. Impact of Vaccination on Laboratory Parameters

**Figure 1:**
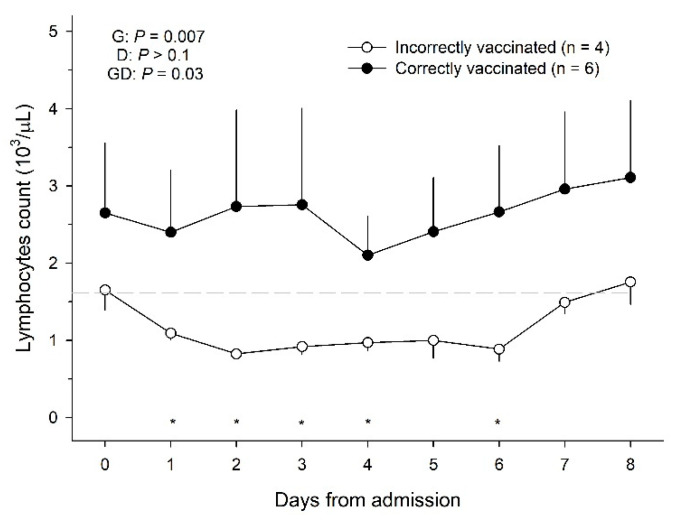
Scatter plot distribution of the evolution of lymphocyte count (±S.E.M.), according to the vaccination status. Mean ± SEM lymphocytes count of correctly vaccinated (n = 6) and incorrectly vaccinated (n = 4) horses for 8 days following admission to the hospital. Probabilities (P) for the effect of group (G), day (D), and group by day interactions (GD) are shown; an asterisk (*) indicates a significant difference (*p* < 0.05) in the value of lymphocytes between the two groups (correctly vaccinated vs. incorrectly vaccinated) in a given day.

Upon admission, lymphopenia was observed in all incorrectly vaccinated horses (4/4), whereas only three of the correctly vaccinated horses exhibited this condition (3/6). Vaccination status was associated with a higher (*p* = 0.02; unpaired *t*-test) lymphocyte count (1.51 ± 0.30 cells/µL) within 24 h of admission compared with incorrectly vaccinated horses (1.08 ± 0.15 cells/µL). The general linear model of variance with repeated measures revealed a significant association of vaccination status and lymphocyte count over the first week after admission (*p* = 0.03; Figure 1). Furthermore, there was a significant interaction between vaccination status and time (day) after admission on the lymphocyte count (*p* = 0.03; Figure 1). This interaction resulted from a gradual reduction in the lymphocyte count in the incorrectly vaccinated group within the first 2 days after admission compared to a constant count in the correctly vaccinated horses. Overall, correctly vaccinated horses had a higher (*p* = 0.007) lymphocyte count than the incorrectly vaccinated group (Figure 1). This difference was significant (*p* < 0.05) between Day 1 to 4 and on Day 6 after admission.

## 4. Discussion

This is the first study to assess the lymphocyte count in a homogeneous population affected by an outbreak of EHM and its relationship with the vaccination status and severity of the disease. The results showed that lymphopenia was the most frequent finding in the population admitted to the VTH affected by EHM, and vaccination status was linked to a higher count of lymphocytes. The results about lymphopenia are consistent with other published EHV-1 studies [4,17]. Lymphopenia is the consequence of a nonspecific acute inflammatory process, but additionally, the EHV-1-infected T cell lymphocytes undergo programmed cell death, to impede the replication and release of the viral pathogen [30,31,32]. This apoptosis of T lymphocytes then leads to lymphopenia and immunosuppression about 2 to 7 days after the viremia [31]. Lymphopenia was already described in different equine viral diseases, such as EHV-1 [4] and equine coronavirus [20,33,34], but no association was established between lymphopenia and the severity of the disease. In human medicine, the level of lymphopenia on admission in patients affected by Herpes zoster (HZ), a reactivated form of human Herpesvirus-3 in adulthood, has been described as a risk factor for prognosis, among others [35]. Recent studies performed on the severe acute respiratory syndrome coronavirus 2 (SARS-CoV-2) caused by COVID-19 have shown that approximately 85% of severely ill patients with COVID-19 suffered from lymphopenia, and this condition has been associated with increased disease severity [36,37,38].

However, to date, extensive information on lymphopenia and its relationship with EHM prognosis is not available. In the current work, the level of lymphopenia was not related to a worse prognosis, as horses with lower lymphocyte counts did not have a longer hospitalization time, and all horses survived. The low number of horses included in the analysis could be a limitation of the study; thus, further research is warranted on this possible prognostic marker.

All the horses evaluated in this study were vaccinated with an inactivated live vaccine. Some studies showed that this type of vaccine protects against EHV-1-induced abortion, reduces the severity of respiratory disease, and minimizes the risk of shedding by decreasing both the viral load and the duration of virus excretion [39,40]. But traditionally, inactivated vaccines have been in vogue, as they are known to produce only a humoral immune response, with limited clinical and virological protection and a requirement for repeated vaccination [41]. Over the years, numerous vaccines have been developed to prevent and control infections and diseases caused by EHV-1. The findings of some studies on this topic are often contradictory, with some of them lacking sufficient scientific rigor [15], highlighting the widely recognized value and limitations of current commercial vaccines [14]. Moreover, no published data currently support the efficacy of any vaccine in providing protection against the neurological form of the disease [39].

In our research, the results showed that correctly vaccinated horses had a lymphocyte count within the normal reference values, but they still had a longer hospitalization time. It has been shown that vaccinated horses were more likely to be euthanized (71%) compared to unvaccinated horses (10%) during an EHM outbreak [17]; and an association between number of vaccines received by the same horse and EHM development has been suggested by Rick W. Henninger and co-workers [12], who demonstrated that horses who had received > 2 vaccines had a 40.5% probability of developing EHM, while those who had received ≤ 2 had only a 23.5%. Furthermore, Traub Dargatz and co-workers [29] showed that horses administered the EHV-1 with the vaccine within 5 weeks prior to EHV-1 exposure were more likely to develop EHM than those vaccinated more than 5 weeks prior to exposure, suggesting a unique window of vulnerability with a higher risk of developing EHM for recently vaccinated horses. Therefore, even though the possible association of EHM with vaccinated horses has been questioned in multiple studies, no clear consensus has yet been reached [10,29]. The recent meta-analysis on EHV-1 [16] explains that even if the vaccine reduces the incidence of EHV-1 infection, there is no evidence to suggest it reduces the development or severity of EHM, and although some studies hypothesize that the vaccination increases the occurrence of EHM [17,42], considerable controversy surrounds the EHV-1 vaccination, as other research contradicts this idea [1,39]. The hypothesis that EHV-1 vaccination status could be linked to a worse prognosis could be related to that vaccine administration stimulates the immune system, triggering the production of antibodies and other immune defenses, thereby generating a specific immune response [43]. This would provoke a greater immune response that could trigger a more systemic inflammatory reaction, leading to more severe illness and a worse prognosis. In a recent study aimed at defining the immune parameters associated with clinical EHM in an equine model, it was observed that horses that develop EHM exhibit a diminished IFN-α response along with increased production of IL-10 and TGF-β, suggesting a dysregulated innate immune response [44]. The level of systemic inflammatory response and its associated complications has been shown in recent studies as a determining factor for the prognosis of the disease [17,45]. The small number of horses included in the current study made it impossible to verify this hypothesis. However, the potential effect of the vaccination on the immune system and its relationship with EHM development strongly advocates for amplified research on this topic.

On the other hand, based on the results of the current study, correctly vaccinated horses may benefit from an early diagnosis and specific treatment to improve outcomes, but that should not be interpreted as a factor that discourages vaccination. The notable decrease in viral shedding observed in vaccinated horses supposes a rational basis for administering booster vaccinations to non-exposed horses at risk of infection [46].

Furthermore, as demonstrated by the results of the current study, correctly vaccinated horses, despite requiring longer hospitalization, did not show a reduction in their lymphocyte count. Their lymphocyte levels remain higher at the onset of disease, and the difference between both groups was significant between several days during the first week of hospitalization. The memory cells obtained by the vaccination could result in a humoral response that is more prepared and effective, and that can neutralize the pathogen faster and consequently cause less severe lymphopenia. Humoral and cellular responses play a crucial role in the development of EHM [47]; therefore, the effects of vaccination and the immune system response should be the focus of future research lines.

An important consideration to take into account regarding the results of this study is that the treatment of the horses analyzed included corticosteroids; however, no horse received this therapy before admission. The dosage administered was never above 0.1 mg/kg IV every 24 h of dexamethasone, nor was this dose maintained for more than 3 days. Corticosteroids are widely used in the treatment of various diseases involving inflammatory, neoplastic, or immunological processes. Several immunosuppressive effects of these agents have been observed in both humans and animals, including temporary lymphopenia [48]. Yet, it has been shown that corticosteroids can affect the lymphocyte counts in horses [49], which could bring a potential limitation to this study. However, it is important to note that adverse effects of corticosteroids appear to be associated with the dose and duration of treatment; and low doses, such as ≤0.1 mg/kg IV, are generally considered less likely to induce significant immunosuppression [48,50,51,52,53]. In fact, recently, a study evaluating the effect of corticosteroid therapy on the immune system of horses vaccinated against EHV-1 suggested that the effects of dexamethasone on the equine immune system are complex and not yet fully understood. It argues that different dosage regimens of dexamethasone or other corticosteroids might have varying effects on the antibody response in horses, which have not been sufficiently investigated [54]. In humans, it has been demonstrated that the impact of corticosteroids on lymphocytes is largely time- and dose-dependent [55,56]. In this study, in addition, corticosteroid therapy was administered in both groups, so its use during this outbreak would not bias the effect of vaccination status on lymphocyte count. However, since corticosteroids are a commonly used therapy in horses with vasculitis, such as those affected by EHM, future studies on the impact of this treatment on these animals, as well as its effects on the immune system, represent an important line of research.

This study represents a unique opportunity to assess the impact of vaccination on lymphocyte counts in horses affected by EHM during a natural EHV-1 outbreak, as well as its progression during the first week of the disease. However, it has limitations that should be considered when interpreting the results and designing future studies to further explore the effects of EHV-1 vaccination in horses. The most significant limitation of this study is that the conclusions are based on a single type of vaccine, studied in a small number of animals within a specific horse population. Additionally, since this study involved animals affected during a natural outbreak, the timing of the last vaccine dose was not the same for all individuals, nor were the number of doses received within different groups. It would have been valuable to have a larger sample size that allows for the replication of this study using other commercial vaccines available, as well as to divide the animals into three groups based on whether they had received a complete vaccination schedule, an incomplete vaccination schedule, or no vaccination at all. This would have allowed for a more robust control group, a clearer assessment of the impact of different vaccination statuses, and the ability to compare results across different vaccine types. Lastly, monitoring lymphocyte evolution until the complete recovery of the animals would have been interesting and should ideally be included in future experimental studies.

Therefore, the results of this study should be interpreted within the specific context of this outbreak and the type of vaccine used. Future research utilizing alternative vaccine formulations is essential to assess whether these findings can be generalized to other types of EHV-1 vaccines. Expanding investigations to include a broader range of vaccine types and larger, more diverse populations will be critical for developing more effective and comprehensive strategies to manage and prevent EHV-1 natural infections.

## 5. Conclusions

Lymphopenia was the most frequent finding in horses infected with EHV-1 in the studied population. Correct vaccination prior to an EHM outbreak was associated with a prolonged hospitalization. Correctly vaccinated horses exhibited higher lymphocyte counts during the first 24 h post-admission compared to incorrectly vaccinated horses, a trend that persisted throughout the first week of hospitalization. The pivotal role of the immune system in modulating the severity of EHM outbreaks underscores the importance of further research in this area.

## Figures and Tables

**Table 1:** Characteristics of horses affected by equine herpesvirus 1-induced myeloencephalopathy (EHM) and the impact of vaccination status on disease severity and clinical parameters.

EHM Outbreak Parameters	Vaccination Status Against EHV-1 Prior to Outbreak		*p* Value
	Correctly Vaccinated	Incorrectly Vaccinated	
Number of affected horses (n)	6	4	-
Age years old	10 ± 2	8.2 ± 1.5	NS
Gender (female/gelding ratio)	3/3	1/4	NS
Fever (%)	50.0	0.0	NS
Lymphopenia at admission (%)	50.0	100.0	NS
Lymphocytes at admission (×10^3^ cells/µL)	1.51 ± 0.30	1.08 ± 0.15	0.02
Systemic complications (%)	33.3	0.0	NS
Median ataxia grade (Min.–Max.)	3 (0–4)	3 (2–3)	NS
Mean hospitalization time (days)	15.5 ± 1.2	12.5 ± 1.2	0.01

NS (not significant).

## Data Availability

The original contributions presented in this study are included in the article. Further inquiries can be directed to the corresponding author.

## Supplementary Materials

The following supporting information can be downloaded at: https://www.mdpi.com/article/10.3390/ani15071019/s1. Therapeutic management for patients.

## Abbreviations

COVID-19	Coronavirus 2019
CNS	central nervous system
EHM	equine herpesvirus myeloencephalopathy
EHV-1	equine herpesvirus type 1
FEI	International Equestrian Federation
PBMCs	peripheral blood mononuclear cells
PCR	polymerase chain reaction
SARS-CoV-2	severe acute respiratory syndrome coronavirus 2
SIRS	systemic inflammatory response syndrome
VTH	veterinary teaching hospital
