# Supplementary material for: The Effect of Vaccination Status on Total Lymphocyte Count in Horses Affected by Equine Herpes Virus-1 Myeloencephalopathy
Source: Animals (Basel). 2025 Apr 1;15(7):1019. doi: 10.3390/ani15071019 (PMC11987750; doi:10.3390/ani15071019)
Supplement: Supplementary file 1 [file animals-15-01019-s001.zip › animals-3510935-supplementary.pdf]

Supplementary Material

Supplementary Material S1

S-1 Therapeutic management for patients

Stall confinement was initiated during the first week of the outbreak for patients with ataxia grades ≥ 3/5, whereas those with ataxia grades < 3/5 were prescribed 3 10-minute walks per day. The horses were physically examined in their stables at least 6 times per day. A neurological examination was performed daily on all horses to adapt treatment to each patient's progression, according to the recommendations established for EHM.[7]

The medical therapy used in horses included: flunixin meglumine (Nixyvet, Divasa-Farmavic), (1.1 mg/kg IV q 24h), dexamethasone (Caliercortin, Calier), (0.1 mg/kg IV q24h for 3 days), dimethyl sulfoxide (Dimethyl sulfoxide, Fagron Ibérica), (DMSO, 1 gm/kg IV q24h for 3 days), valacyclovir (Valacyclovir, TecniGen), (30 mg/kg every 8 hours orally lasting 7 consecutive days), diazepam (Valium, Atnahs Pharma), (0,05 mg/kg IV) in patients with seizures, Ringer's lactate (Lactate-RingerVet, B. Braun) compensated with calcium (Calcium Injectable, Labiana Life Sciences) and KCl (Potassium chloride Braun). Fluid therapy was complemented with 5% glucose solution (GlucosaVet, Braun) to ensure a correct energy balance. Lastly, omeprazol (Gastrogard, Boehringer Ingelheim), (2 mg/kg c/24h PO) and misoprostol (Cytotec, Pfizer) (5 mcg/kg c/12h PO) were used as digestive ulcer prevention therapy.Once the patients came out of the critical phase of the disease, rehabilitation exercises began. This therapy involved: proprioception exercises, core work and muscle electrostimulation (EMS).

[7] Lunn DP, Davis-Poynter N, Flaminio MJBF, Horohov DW, Osterrieder K, Pusterla N, et al. Equine herpesvirus-1 consensus statement. J Vet Intern Med 2009;23:450–61. https://doi.org/10.1111/j.1939-1676.2009.0304.x.
